# Congenital Anomalies in Infants in Fukushima from 2011 to 2014: The Japan Environment and Children’s Study

**DOI:** 10.31662/jmaj.2022-0087

**Published:** 2022-12-26

**Authors:** Hidekazu Nishigori, Keiya Fujimori, Mitsuaki Hosoya, Toshie Nishigori, Tsuyoshi Murata, Hyo Kyozuka, Yuka Ogata, Akiko Sato, Kosei Shinoki, Seiji Yasumura, Koichi Hashimoto, The Japan Environment and Children’s Study Group

**Affiliations:** 1 Fukushima Regional Center for the Japan Environment and Children’s Study, Fukushima, Japan; 2 Department of Development and Environmental Medicine, Fukushima Medical Center for Children and Women, Fukushima Medical University Graduate School of Medicine, Fukushima, Japan; 3 Department of Obstetrics and Gynecology, Fukushima Medical University School of Medicine, Fukushima, Japan; 4 Department of Pediatrics, Fukushima Medical University School of Medicine, Fukushima, Japan; 5 Department of Public Health, Fukushima Medical University School of Medicine, Fukushima, Japan

**Keywords:** congenital anomalies, infants, Fukushima, The Japan Environment and Children’s Study

## Abstract

**Introduction:**

This study aimed to assess congenital anomalies among infants from 2011 to 2014 in Fukushima and compare the assessment with that from other geographical regions in Japan.

**Methods:**

We used the dataset of the Japan Environment and Children’s Study (JECS), which is a nationwide prospective birth cohort study. For the JECS, participants were recruited through 15 regional centers (RC), including Fukushima. Pregnant women were recruited between January 2011 and March 2014. The Fukushima RC recruited all municipalities in the Fukushima Prefecture, from where we compared congenital anomalies in infants from the Fukushima RC to those in the infants from 14 other RCs. Crude and multivariate logistic regression analyses were also performed, with the multivariate logistic regression analysis being adjusted for maternal age, maternal body mass index (kg/m^2^), infertility treatment, multiple pregnancies, maternal smoking, maternal alcohol consumption, pregnancy complications, maternal infection, and infant sex.

**Results:**

In the Fukushima RC, 12,958 infants were analyzed, and 324 infants were diagnosed with major anomalies (2.50%). In the remaining 14 RCs, 88,771 infants were analyzed and 2,671 infants were diagnosed with major anomalies (3.01%). Crude logistic regression analysis demonstrated that the odds ratio for the Fukushima RC was 0.827 (95% confidence interval, 0.736-0.929) using the other 14 RCs as a reference. Multivariate logistic regression analysis also demonstrated that the adjusted odds ratio was 0.852 (95% confidence interval, 0.757-0.958).

**Conclusions:**

Fukushima Prefecture was found not to be an area at high risk for the occurrence of congenital anomalies in infants compared nationwide in Japan from 2011 to 2014.

## Introduction

The Japan Environment and Children’s Study (JECS) is a nationwide prospective birth cohort study of 100,000 pairs of parents and offspring funded by the Ministry of the Environment of Japan ^(1), (2)^. Pregnant women were recruited between January 2011 and March 2014 at 15 regional centers (RCs) including Fukushima Prefecture.

On March 11^th^, 2011, the Fukushima Prefecture was struck by the Great East Japan Earthquake which was closely followed by the Tokyo Electric Power Company’s Fukushima Daiichi Nuclear Power Plant accident. Since the disaster, the Fukushima Prefecture has been surrounded by rumors about the potential effects of radiation on pregnancies and infants ^(3), (4)^. This is because it was reported that the incidence of infants being born with congenital anomalies in the surrounding region was increased after the Chernobyl nuclear power plant accident ^(5), (6), (7)^. However, a previous survey in the Fukushima Prefecture suggests that the occurrence of congenital anomalies among infants did not increase ^(8)^. Furthermore, another survey in the Fukushima Prefecture that examined the external radiation dose received by pregnant women also did not reveal a relationship between external radiation dose and any increases in the occurrence of congenital anomalies among their infants ^(9)^. However, no statistical analysis was made comparing the results of these studies with those of studies outside the geographical regions of the Fukushima Prefecture in Japan.

The JECS was continued after the accidents in Fukushima Prefecture, and one of the outcomes of this study is the presence of congenital anomalies among infants. This study aimed to assess congenital anomalies among infants in the Fukushima Prefecture using JECS data from 2011 to 2014 and compare it with that from other geographical regions in Japan.

## Materials and Methods

### Design and participants

**Figure 1.**
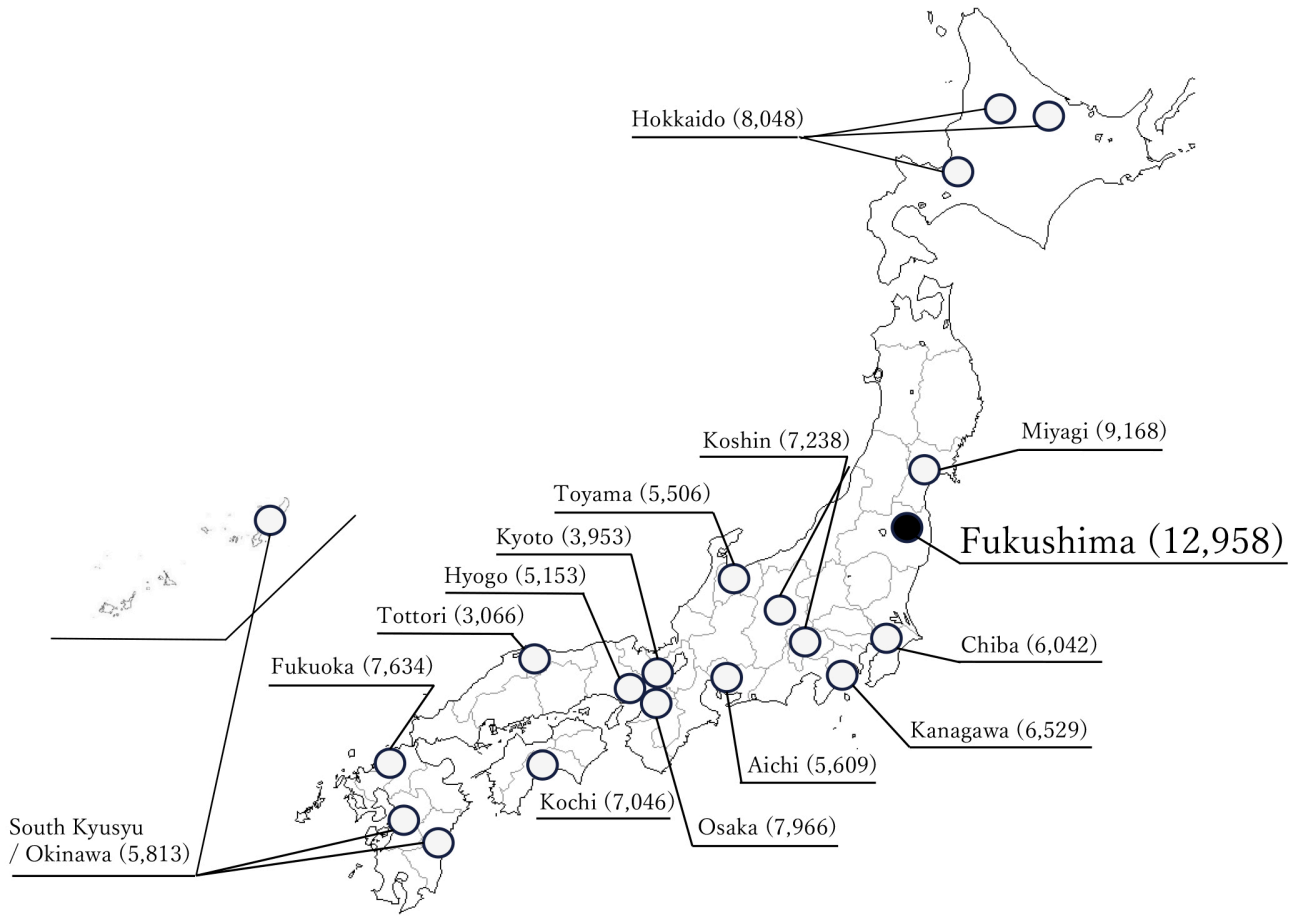
The 15 regional centers of Japan Environment and Children’s Study (JECS) and infants in this study. Figures in parentheses indicate the number of participants.

**Figure 2.**
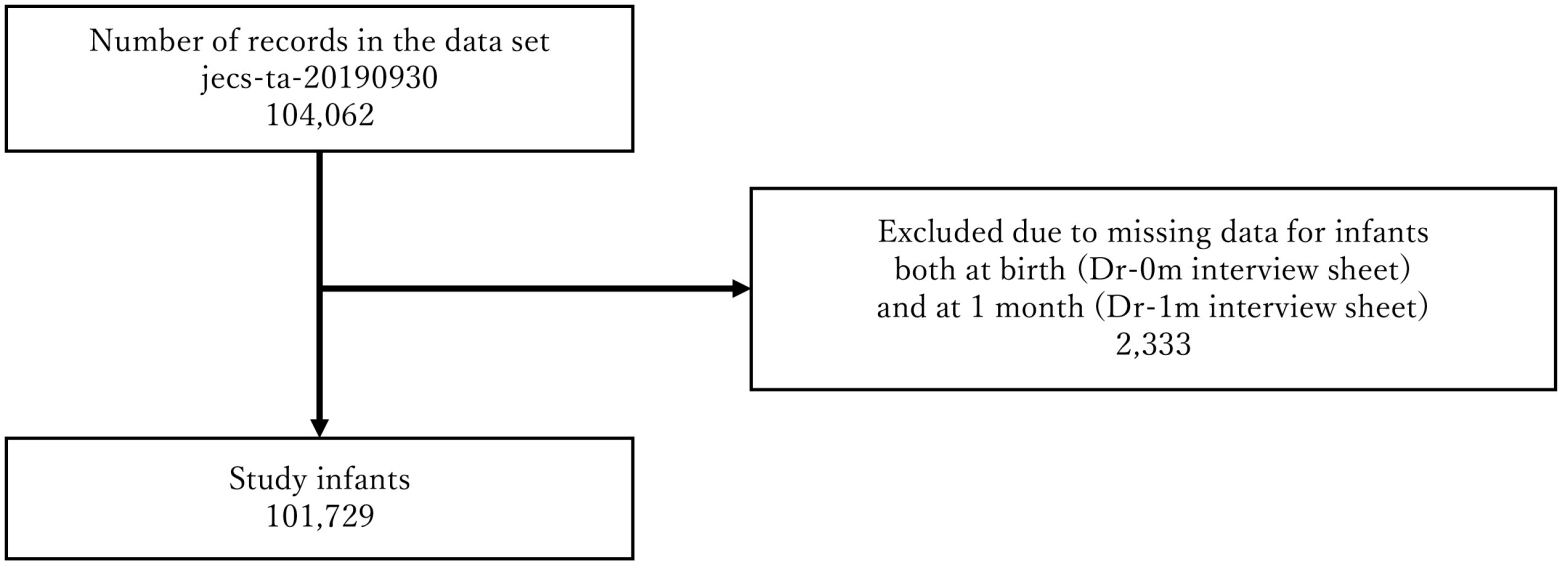
The flow chart for research participant selection.

The JECS protocol has been published elsewhere ^(1), (2)^. This study was conducted according to the guidelines laid down in the Declaration of Helsinki. The JECS protocol was reviewed and approved by the Ministry of the Environment’s Institutional Review Board on Epidemiological Studies (no. 100910001) and the Ethics Committees of all participating institutions. JECS recruitment of pregnant women occurred nationwide between January 2011 and March 2014. For the JECS, participants were recruited through 15 RCs located in Hokkaido, Miyagi, Chiba, Kanagawa, Koshin, Toyama, Aichi, Kyoto, Osaka, Hyogo, Tottori, Kochi, Fukuoka, South Kyushu/Okinawa, and Fukushima (Figure 1). Written informed consent was obtained from all participants. The infants included live births, stillbirths, spontaneous abortion and induced abortion. The present study used the jecs-ta-20190930 dataset, which was revised in June 2021. Of the 104,062 records in these datasets, records from 101,729 infants and their mothers were analyzed (Figure 2).

### Participants of Fukushima regional center

Recruitment of pregnant women in the Study Area within Fukushima Prefecture, which included Fukushima City, Minami Soma City, and Futaba County, was initiated on January 31^st^, 2011 with the co-operating health care providers. On March 11^th^, 2011, the Great East Japan Earthquake struck the Fukushima Prefecture. As a result of the disaster, the Study Area in the Fukushima Prefecture was expanded to include all 59 municipalities across the Fukushima Prefecture in October 2012, where recruitment continued until the end of the recruitment period in March 2014 ^(10)^. Infants whose mothers were enrolled in the Fukushima RC at recruitment (during pregnancy) were classified as participants from the Fukushima RC.

### Participants of the other 14 regional centers

Participants were enrolled at the time of recruitment in 14 RCs outside the Fukushima Prefecture. The 14 RCs are located in Hokkaido, Miyagi, Chiba, Kanagawa, Koshin, Toyama, Aichi, Kyoto, Osaka, Hyogo, Tottori, Kochi, Fukuoka, and Southern Kyushu/Okinawa.

Infants whose mothers were enrolled in each of the various RCs at recruitment (during pregnancy) were analyzed as participants from their respective RCs.

### Congenital anomalies

The present analyses focused on a list of 61 congenital anomalies in accordance with the protocol paper of JECS ^(11)^. Physicians diagnosed these anomalies immediately after delivery and during the first month at a regular check-up. Congenital anomalies reported either at delivery or at one-month data collection were analyzed for this study ^(11)^. The data set provided omitted omphalocele in the data collected after one month of delivery. Therefore, for omphalocele, only information at delivery was analyzed.

### Statistical analysis and covariables

The frequency of congenital anomalies of infants between Fukushima RC and the other 14 RCs was analyzed using the chi-square test. We also used crude and multivariate logistic regression analyses to obtain odds ratios (ORs) and 95% confidence intervals (CIs) for the Fukushima RC, using the other 14 RCs as a reference.

Multivariate logistic regression analysis was adjusted for maternal age at delivery, maternal body mass index (kg/m^2^) before pregnancy, treatment for infertility, multiple pregnancy, maternal smoking during pregnancy, maternal alcohol consumption during pregnancy, pregnancy complications, maternal infection, and infant sex ^(2)^. These covariates have been reported as factors associated with the development of congenital anomalies ^(12)^. The answer ‘no’ was analyzed as a single item for each confounder. Pregnancy complications included hypertension, hyperthyroidism, hypothyroidism, diabetes, autoimmune disease, cardiac disease, renal disease, hepatitis, cerebral infarction, intracerebral hemorrhage, epilepsy, hematological disease, malignant tumor, psychiatric disease, neurological disease, and thrombosis. With the results of these outcomes not being presented, maternal infection included toxoplasmosis, syphilis, HBV hepatitis, rubella, cytomegalovirus infection, and herpes simplex virus infection. The same multiple logistic regression model was applied for each congenital anomaly, but many of the congenital anomalies were rare and completely separated data points. Therefore, the results of these outcomes were not presented.

All statistical analyses were performed using SAS statistical software (version 9.4; SAS Institute Inc., Cary, NC, USA).

## Results

Of the 104,062 records in this dataset, records from 101,729 infants were analyzed (Figure 2). Table 1 shows the characteristics of the participants.

**Table 1.** Participant Characteristics.

		Total		Fukushima RC		Other 14 RCs	
		n = 101,729		n = 12,958		n = 88,771	
		n	%	n	%	n	%
Maternal age at delivery	≤24	10174	10.0	1595	12.3	8579	9.7
	25-34	63846	62.8	8349	64.4	55497	62.5
	≥35	27684	27.2	3012	23.2	24672	27.8
	No answer	25	0.02	2	0.02	23	0.03
						
Maternal BMI (kg/m^2^) before pregnancy	<18.5	16419	16.1	1884	14.5	14535	16.4
	18.5≤-<25.0	74104	72.8	9411	72.6	64693	72.9
	≥25.0	10965	10.8	1653	12.8	9312	10.5
	No answer	241	0.2	10	0.1	231	0.3
					
Infertility treatment	No	94121	92.5	12262	94.6	81859	92.2
	Yes	7007	6.9	690	5.3	6317	7.1
	No answer	601	0.6	6	0.1	595	0.7
					
Multiple pregnancy	No	99741	98.1	12707	98.1	87034	98.0
	Yes	1988	2.0	251	1.9	1737	2.0
					
Maternal smoking during pregnancy	No	94241	92.6	12270	94.7	81971	92.3
	Yes	4765	4.7	557	4.3	4208	4.7
	No answer	2723	2.7	131	1.0	2592	2.9
					
Maternal alcohol consumption during pregnancy	No	89443	87.9	11551	89.1	77892	87.7
	Yes	9848	9.7	1284	9.9	8564	9.7
	No answer	2438	2.4	123	1.0	2315	2.6
					
Pregnancy complication	No	84309	82.9	11698	90.3	72611	81.8
	Yes	15140	14.9	1138	8.8	14002	15.8
	No answer	2280	2.2	122	0.9	2158	2.4
					
Maternal infection	No	98811	97.1	12740	98.3	86071	97.0
	Yes	2918	2.9	218	1.7	2700	3.0
					
Birth	Live birth	100054	98.4	12803	98.8	87251	98.3
	Stillbirth	382	0.4	70	0.5	312	0.4
	Spontaneous abortion or induced abortion	1254	1.2	85	0.7	1169	1.3
	No answer	39	0.04	0	0.00	39	0.04
					
Sex of infants	Boy	51711	50.8	6674	51.5	45037	50.7
	Girl	49033	48.2	6234	48.1	42799	48.2
	Unknown	535	0.5	39	0.3	496	0.6
	No answer	450	0.4	11	0.1	439	0.5

Abbreviations: regional center (RC), body mass index (BMI) Pregnancy complications included hypertension, hyperthyroidism, hypothyroidism, diabetes, autoimmune disease, cardiac disease, renal disease, hepatitis, cerebral infarction, intracerebral hemorrhage, epilepsy, hematological disease, malignant tumor, psychiatric disease, neurological disease, thrombosis, and others. Maternal infection included toxoplasmosis, syphilis, HBV hepatitis, rubella, cytomegalovirus infection, herpes simplex virus infection.

In the Fukushima RC, 12,958 infants were analyzed, and 324 infants were diagnosed with major anomalies (2.50%). In the remaining 14 RCs, 88,771 infants were analyzed, and 2,671 infants were diagnosed with major anomalies (3.01%) (Table 2). Crude logistic regression analysis demonstrated that the crude odds ratio (COR) for the Fukushima RC was 0.827 (95% CI, 0.736-0.929) using the other 14 RCs as reference (Table 3). Multivariate logistic regression analysis also demonstrated that the adjusted odds ratio (AOR) for the Fukushima RC was 0.852 (95% CI, 0.757-0.958) using the other 14 RCs as reference (Table 3).

**Table 2.** Number and Prevalence of Congenital Anomalies.

		Total		Fukushima RC		Other 14RCs		Chi-square test
		n	per 10,000	n	per 10,000	n	per 10,000	p
		101,729		12,958		88,771		
Major anomalies		2995	294.4	324	250.0	2671	300.9	0.001
*Central nervous system*		312	30.7	37	28.6	275	31.0	0.64
	*Neural tube defect*	88	8.7	8	6.2	80	9.0	0.30
	Anencephaly	32	3.1	2	1.5	30	3.4	0.42
	Encephalocele	22	2.2	3	2.3	19	2.1	0.75
	Myelomeningocele/Spina bifida	36	3.5	4	3.1	32	3.6	1.00
	Hydrocephaly	89	8.7	10	7.7	79	8.9	0.67
	Microcephaly	38	3.7	2	1.5	36	4.1	0.22
	Holoprosencephaly	43	4.2	5	3.9	38	4.3	0.83
	Craniotabes	69	6.8	13	10.0	56	6.3	0.13
	Agenesis of corpus callosum	23	2.3	1	0.8	22	2.5	0.35
*Eye*		50	4.9	10	7.7	40	4.5	0.12
	Anophthalmos/microphthalmos	22	2.2	2	1.5	20	2.3	1.00
	Congenital cataract	30	2.9	9	6.9	21	2.4	0.01
*Ear*		71	7.0	11	8.5	60	6.8	0.49
	Congenital aural atresia	40	3.9	6	4.6	34	3.8	0.67
	Cryptotia	33	3.2	6	4.6	27	3.0	0.30
*Oro-facial cleft*		253	24.9	32	24.7	221	24.9	0.97
	Cleft palate	54	5.3	7	5.4	47	5.3	0.96
	Cleft lip with or without palate	198	19.5	26	20.1	172	19.4	0.83
	Facial cleft	9	0.9	0	0.0	9	1.0	0.61
*Respiratory system*		52	5.1	7	5.4	45	5.1	0.88
	Intralobar sequestration	5	0.5	1	0.8	4	0.5	0.49
	Congenital cystic adenomatoid malformation	15	1.5	3	2.3	12	1.4	0.43
	Pulmonary hypoplasia	34	3.3	4	3.1	30	3.4	1.00
*Congenital heart disease*		1230	120.9	110	84.9	1120	126.2	<.0001
*Cardiac arrhythmia*		106	10.4	7	5.4	99	11.2	0.06
*Abdominal wall defects*		261	25.7	17	13.1	244	27.5	0.003
	Omphalocele	243	23.9	13	10.0	230	25.9	0.001
	Gastroschisis	21	2.1	4	3.1	17	1.9	0.33
*Digestive system*		140	13.8	20	15.4	120	13.5	0.58
	Esophageal atresia with or without fistula	23	2.3	6	4.6	17	1.9	0.06
	Duodenal atresia/stenosis	19	1.9	2	1.5	17	1.9	1.00
	Intestinal atresia/stenosis	18	1.8	3	2.3	15	1.7	0.49
	Anorectal atresia/stenosis	46	4.5	7	5.4	39	4.4	0.61
	Diaphragmatic hernia	43	4.2	3	2.3	40	4.5	0.26
*Urinary system*		265	26.0	32	24.7	233	26.2	0.75
	Congenital hydronephrosis	217	21.3	26	20.1	191	21.5	0.74
	Cystic kidney	37	3.6	4	3.1	33	3.7	1.00
	Renal agenesis	13	1.3	2	1.5	11	1.2	0.68
	Bladder exstrophy	3	0.3	1	0.8	2	0.2	0.34
*Genital system*		73	7.2	4	3.1	69	7.8	0.06
	Hypospadias	66	6.5	4	3.1	62	7.0	0.10
	Indeterminate sex	9	0.9	1	0.8	8	0.9	1.00
*Limb*		273	26.8	50	38.6	223	25.1	0.01
	*Polydactyly*	184	18.1	39	30.1	145	16.3	0.001
	Polydactyly of fingers	111	10.9	19	14.7	92	10.4	0.17
	Polydactyly of toes	89	8.7	23	17.7	66	7.4	0.0002
	*Syndactyly*	135	13.3	24	18.5	111	12.5	0.08
	Syndactyly of fingers	47	4.6	10	7.7	37	4.2	0.08
	Syndactyly of toes	104	10.2	16	12.3	88	9.9	0.42
	*Cleft hand or foot*	10	1.0	2	1.5	8	0.9	0.37
	Cleft hand	5	0.5	1	0.8	4	0.5	0.49
	Cleft foot	7	0.7	2	1.5	5	0.6	0.22
*Skeletal dysplasia*		12	1.2	0	0.0	12	1.4	0.38
	Thanatophoric dysplasia	6	0.6	0	0.0	6	0.7	1.00
	Acrodysostosis, not specificied	6	0.6	0	0.0	6	0.7	1.00
*Chromosomal*		208	20.4	22	17.0	186	21.0	0.35
	Down syndrome	153	15.0	14	10.8	139	15.7	0.18
	Trisomy 18	45	4.4	6	4.6	39	4.4	0.90
	Trisomy 13	8	0.8	2	1.5	6	0.7	0.27
	Turner syndrome	6	0.6	1	0.8	5	0.6	0.56
*Others*								
	Ablepharon	13	1.3	0	0.0	13	1.5	0.39
	Epidermolysis bullosa	15	1.5	2	1.5	13	1.5	1.00
	Developmental dysplasia of the hip	14	1.4	3	2.3	11	1.2	0.41
	Congenital multiple arthrogryposis	8	0.8	2	1.5	6	0.7	0.27
	Floppy infant	16	1.6	2	1.5	14	1.6	1.00
	Conjoined twins	4	0.4	1	0.8	3	0.3	0.42
	Amniotic band constriction	9	0.9	4	3.1	5	0.6	0.02
Minor anomalies								
	Microtia	40	3.9	1	0.8	39	4.4	0.052
	Low set ears	93	9.1	8	6.2	85	9.6	0.23
	Natal teeth	64	6.3	9	6.9	55	6.2	0.75
	Inguinal hernia	112	11.0	11	8.5	101	11.4	0.35
	Undescended testis/cryptorchidism	307	30.2	25	19.3	282	31.8	0.02
	Enlarged clitoris	12	1.2	2	1.5	10	1.1	0.66
	Abnormal Vaginal Opening	3	0.3	0	0.0	3	0.3	1.00
	Hemangioma	756	74.3	80	61.7	676	76.2	0.07

Abbreviations: (RC), regional center

**Table 3.** Crude and Multivariate Logistic Regression Analysis Results for Fukushima RC Using the Other 14 RCs as a Reference.

			COR	95%CI			p	AOR	95%CI			p
Major anomalies			0.827	0.736	-	0.929	0.001	0.852	0.757	-	0.958	0.01
*Central nervous system*			0.922	0.653	-	1.299	0.64	0.974	0.689	-	1.377	0.88
	*Neural tube defect*		0.685	0.331	-	1.417	0.31	0.709	0.341	-	1.472	0.36
		Anencephaly	0.457	0.109	-	1.911	0.28	0.475	0.113	-	1.997	0.31
		Encephalocele	1.082	0.320	-	3.656	0.90	-				
		Myelomeningocele/Spina bifida	0.856	0.303	-	2.422	0.77	0.864	0.303	-	2.461	0.78
	Hydrocephaly		0.867	0.449	-	1.674	0.67	0.966	0.498	-	1.876	0.92
	Microcephaly		0.381	0.092	-	1.581	0.18	-				
	Holoprosencephaly		0.901	0.355	-	2.290	0.83	-				
	Craniotabes		1.591	0.870	-	2.910	0.13	-				
	Agenesis of corpus callosum		0.311	0.042	-	2.310	0.25	-				
*Eye*			1.713	0.857	-	3.427	0.13	-				
	Anophthalmos/microphthalmos		0.685	0.160	-	2.931	0.61	-				
	Congenital cataract		2.937	1.345	-	6.415	0.01	-				
*Ear*			1.256	0.660	-	2.390	0.49	-				
	Congenital aural atresia		1.209	0.508	-	2.881	0.67	-				
	Cryptotia		1.523	0.629	-	3.689	0.35	-				
*Oro-facial cleft*			0.992	0.684	-	1.438	0.97	0.997	0.686	-	1.448	0.99
	Cleft palate		1.020	0.461	-	2.258	0.96	-				
	Cleft lip with or without palate		1.036	0.686	-	1.566	0.87	1.051	0.693	-	1.592	0.82
	Facial cleft		<0.001	<0.001	-	>999.999	0.96	-				
*Respiratory system*			1.066	0.481	-	2.364	0.88	1.037	0.465	-	2.312	0.93
	Intralobar sequestration		1.713	0.191	-	15.325	0.63	-				
	Congenital cystic adenomatoid malformation		1.713	0.483	-	6.071	0.40	-				
	Pulmonary hypoplasia		0.913	0.322	-	2.593	0.86	-				
*Congenital heart disease*			0.670	0.550	-	0.816	<.0001	0.693	0.569	-	0.845	0.0003
*Cardiac arrhythmia*			0.484	0.225	-	1.042	0.06	0.494	0.229	-	1.067	0.07
*Abdominal wall defects*			0.477	0.291	-	0.780	0.003	0.500	0.305	-	0.819	0.01
	Omphalocele		0.387	0.221	-	0.677	0.001	0.404	0.231	-	0.708	0.002
	Gastroschisis		1.612	0.542	-	4.792	0.39	-				
*Digestive system*			1.142	0.711	-	1.834	0.58	1.197	0.743	-	1.929	0.46
	Esophageal atresia with or without fistula		2.419	0.953	-	6.135	0.06	-				
	Duodenal atresia/stenosis		0.806	0.186	-	3.489	0.77	-				
	Intestinal atresia/stenosis		1.370	0.397	-	4.734	0.62	-				
	Anorectal atresia/stenosis		1.230	0.550	-	2.750	0.61	-				
	Diaphragmatic hernia		0.514	0.159	-	1.661	0.27	-				
*Urinary system*			0.941	0.650	-	1.362	0.75	0.966	0.665	-	1.401	0.85
	Congenital hydronephrosis		0.932	0.619	-	1.405	0.74	0.965	0.639	-	1.458	0.87
	Cystic kidney		0.830	0.294	-	2.344	0.73	-				
	Renal agenesis		1.246	0.276	-	5.621	0.77	-				
	Bladder exstrophy		3.426	0.311	-	37.781	0.31	-				
*Genital system*			0.397	0.145	-	1.088	0.07	0.419	0.153	-	1.153	0.09
	Hypospadias		0.442	0.161	-	1.215	0.11	-				
	Indeterminate sex		0.856	0.107	-	6.847	0.88	-				
*Limb*			1.538	1.131	-	2.091	0.01	1.510	1.108	-	2.057	0.01
	*Polydactyly*		1.845	1.295	-	2.629	0.001	1.826	1.277	-	2.609	0.001
		Polydactyly of fingers	1.415	0.863	-	2.320	0.17	-				
		Polydactyly of toes	2.390	1.486	-	3.843	0.0003	-				
	*Syndactyly*		1.482	0.953	-	2.305	0.08	1.456	0.934	-	2.271	0.10
		Syndactyly of fingers	1.852	0.921	-	3.726	0.08	-				
		Syndactyly of toes	1.246	0.731	-	2.123	0.42	1.239	0.724	-	2.118	0.43
	*Cleft hand or foot*		1.713	0.364	-	8.067	0.50	-				
		Cleft hand	1.713	0.191	-	15.325	0.63	-				
		Cleft foot	2.741	0.532	-	14.127	0.23	-				
*Skeletal dysplasia*			<0.001	<0.001	-	>999.999	0.97	-				
	Thanatophoric dysplasia		<0.001	<0.001	-	>999.999	0.97	-				
	Acrodysostosis, not specificied		<0.001	<0.001	-	>999.999	0.97	-				
*Chromosomal*			0.810	0.521	-	1.261	0.35	0.896	0.574	-	1.398	0.63
	Down syndrome		0.690	0.398	-	1.195	0.19	0.773	0.444	-	1.343	0.36
	Trisomy 18		1.054	0.446	-	2.490	0.90	1.158	0.487	-	2.753	0.74
	Trisomy 13		2.284	0.461	-	11.316	0.31	-				
	Turner syndrome		1.371	0.160	-	11.730	0.77	-				
Other											
	Ablepharon		<0.001	<0.001	-	>999.999	0.97	-				
	Epidermolysis bullosa		1.054	0.238	-	4.671	0.94	-				
	Developmental dysplasia of the hip		1.869	0.521	-	6.699	0.34	-				
	Congenital multiple arthrogryposis		2.284	0.461	-	11.316	0.31	-				
	Floppy infant		0.979	0.222	-	4.307	0.98	-				
	Conjoined twins		2.284	0.238	-	21.956	0.47	-				
	Amniotic band constriction		5.482	1.472	-	20.417	0.01	-				
											
Minor anomalies												
	Microtia		0.176	0.024	-	1.278	0.09	0.175	0.024	-	1.275	0.09
	Low set ears		0.645	0.312	-	1.331	0.24	0.705	0.340	-	1.461	0.35
	Natal teeth		1.121	0.554	-	2.269	0.75	1.120	0.550	-	2.277	0.76
	Inguinal hernia		0.746	0.400	-	1.391	0.36	0.777	0.416	-	1.452	0.43
	Undescended testis/cryptorchidism		0.607	0.403	-	0.913	0.02	0.617	0.409	-	0.930	0.02
	Enlarged clitoris		1.371	0.301	-	6.256	0.68	-				
	Abnormal Vaginal Opening		<0.001	<0.001	-	>999.999	0.97	-				
	Hemangioma		0.810	0.642	-	1.022	0.08	-				

Abbreviations: (RC), regional center; (COR), crude odds ratio; (AOR), adjusted odds ratio; (CI), confidence interval Adjusted for maternal age at delivery, maternal body mass index (BMI)(kg/m^2^) before pregnancy, infertility treatment, multiple pregnancy, maternal smoking during pregnancy, maternal alcohol consumption during pregnancy, pregnancy complication, maternal infection, infant sex. For each congenital anomaly, the same multiple logistic regression model was applied, but many of the congenital anomalies were rare and resulted in complete separation of data points. Therefore, the results of these outcomes were not presented.

When each major anomaly was examined, crude logistic regression analysis revealed that the ORs for congenital heart disease (COR; 0.670, 95% CI; 0.550-0.816) and omphalocele (COR; 0.387, 95% CI; 0.221-0.677), using the other 14 RCs as reference, were significantly low in infants from the Fukushima RC. For minor anomalies, undescended testis/cryptoracism (COR, 0.607; 95%CI, 0.403-0.913) was significantly low in infants from the Fukushima RC (Table 3). Multiple logistic regression analysis showed that the ORs for congenital heart disease (AOR, 0.693; 95% CI; 0.569-0.845), omphalocele (AOR, 0.404; 95% CI, 0.231-0.708), and undescended testis/cryptoracism (AOR: 0.617, 95% CI; 0.409-0.930) were significantly low in infants in the Fukushima RC using the other 14 RCs as reference (Table 3).

Crude logistic regression analysis demonstrated that the ORs for congenital cataract (COR; 2.937, 95% CI; 1.345-6.415), polydactyly of toes (COR, 2.390; 95% CI; 1.486-3.843), and amniotic band constriction (COR; 5.482, 95% CI; 1.472-20.417) were significantly high in infants in the Fukushima RC (Table 3). The multiple logistic regression model was applied for congenital cataracts, polydactyly of toes, and amniotic band constriction, but these congenital anomalies separated the data points. Therefore, the results of these outcomes are not presented.

## Discussion

In this study, which included data from the JECS study, Fukushima Prefecture was found to have a significantly lower risk for infant congenital anomalies than that in other regions nationwide in Japan from 2011 to 2014. In other words, the Fukushima Prefecture was not found to be an area with increased risks for the occurrence of infants with congenital anomalies.

Previously, we reported that the frequency of infants with anomalies in Fukushima Prefecture was 2.72%, according to the Pregnancy and Birth Surveys comprising the Fukushima Health Management Survey ^(8)^. This survey collected data to improve obstetrical and prenatal care and support women who were pregnant or delivered infants in the Fukushima Prefecture during and after the Great East Japan Earthquake on March 11^th^, 2011. The participants were pregnant women who received Maternal and Child Health Handbooks between August 2010 and July 2011. Although the conditions of this study were different from those of the JECS, the frequency of congenital anomalies in infants in Fukushima was similar to the frequency of congenital abnormalities of our JECS study (2.50%). The JECS study found a significantly lower risk of congenital heart disease, omphalocele, and undescended testis/cryptorchidism in infants in the Fukushima RC than that in infants from the other 14 RCs.

On the contrary, under the limitation that multiple logistic regression analysis could not demonstrate, and although the reasons for the divergence and feature in the Fukushima RC were unclear, crude logistic regression analysis demonstrated a significantly higher risk of congenital cataract, polydactyly of toes, and amniotic band constriction in infants in the Fukushima RC than in the other 14 RCs. The causes of congenital anomalies are mostly unknown and complicated by various factors, including hereditary disease, multifactorial disease, idiopathic genetic mutations, maternal disease, maternal drug use, mother-to-child transmission, maternal nutrition, and environmental agents ^(12)^.

With regard to congenital heart disease, our results showed that the incidence among infants was significantly lower in Fukushima than in other regions. However, a limitation of our study is the lack of detailed disease information concerning congenital heart disease. While it is not known whether echocardiography was used for evaluation of congenital heart diseases in the participants included in this study, checking for cardiac murmurs by auscultation is the routine screening method for congenital heart disease. In previous studies, Murase et al. reported that the number of operations for complex congenital heart disease in infants increased nationwide after the Fukushima Daiichi Nuclear Power Plant accident, using data from the annual surveys conducted by the Japanese Association for Thoracic Surgery between 2007 and 2014 ^(13)^. However, it should be noted, when looking specifically at the Fukushima Prefecture, that they did not report an increase the number of operations for complex congenital heart disease. Moreover, it has been noted that this paper has an inappropriate evaluation methodology ^(14)^. Contrarily, Hirata et al. reported that there was no increase in the number of patients with congenital heart disease from 2010 to 2013 ^(14)^. They also concluded that the yearly increase in the total number of surgeries following the Fukushima Daiichi Nuclear Power Plant accident in the study of Murase et al. can be explained by a decline in the mortality rate for first-time surgeries for complex cases ^(14)^. Thus, using only the increase in the total yearly number of surgeries to claim the effects of a nuclear disaster on the incidence of congenital heart disease would be far too simplistic and dangerous a proposition ^(14)^.

With regard to undescended testis/cryptorchidism in infants, we found the incidence to be significantly lower in Fukushima than it was in other regions. In a previous study, Murase et al. reported that the discharge rate of cryptorchidism in infants increased nationwide after the Fukushima Daiichi Nuclear Power Plant accident, by using data from the Diagnosis Procedure Combination survey database in Japan from between 2010 and 2015 ^(15)^. However, it should be noted, when looking specifically at the Fukushima Prefecture, that they did not report an increase the discharge rate of cryptorchidism in infants. Moreover, Kojima et al. argued that the study of Murase et al. failed to establish biological plausibility for their hypothesis and glossed over an abundance of evidence and expert opinion to the contrary ^(16)^. Further, when reporting undescended testis/cryptorchidism ^(16)^, numerous factors must be considered, including genetic, environmental, maternal/fetal, and social factors. It has been established that the doses of external and internal radiation exposure in both Fukushima Prefecture and the whole of Japan after the accident were too low to affect undescended testis/cryptorchidism during fetal periods; thus, a putative association between radiation exposure and fetal periods can be theoretically and empirically rejected ^(16)^.

Congenital cataracts were found to be more frequent in infants in the Fukushima RC than in the other 14 RCs. However, the results need to be carefully interpreted because we were unable to perform a multivariate logistic regression analysis with the necessary confounding factors inputted as a complete separation of data points. The causes of congenital cataracts are primarily unknown. They include idiopathic, chromosomal abnormalities, ocular and systemic diseases, metabolic diseases, mother-to-child transmission, malnutrition during pregnancy, and other maternal diseases. Congenital cataracts may also be solitary familial congenital anomalies that are generally inherited in an autosomal dominant fashion ^(17)^. Cataracts can be caused by radiation, either due to a high radiation dose or a low level of radiation exposure over a long time ^(18)^. The International Commission on Radiological Protection recommends an occupational equivalent dose limit of 0.5 Gy for the lens for the prevention of cataracts ^(18)^. However, it is theoretically highly unlikely that radiation is the cause of congenital cataracts in infants, even when in utero exposure is considered, as, according to Yasuda et al., the external exposure-dose of pregnant women in Fukushima Prefecture after the Fukushima Daiichi Nuclear Power Plant accident was ≤5.2 mSv and mostly <2 mSv ^(9)^.

Polydactyly of toes were found to be more frequent in infants in the Fukushima RC than in the other 14 RCs. However, the results need to be carefully interpreted because we were unable to perform a multivariate logistic regression analysis with the necessary confounding factors inputted as a complete separation of data points. The cause of polydactyly in toes is usually transmitted as autosomal dominant. Furthermore, there is some evidence that environmental factors play a role in isolated polydactyly cases that are not familial; it occurs more frequently in mothers with maternal diabetes that have upper respiratory tract infections in the first three months of pregnancy and in children whose mothers had a history of epilepsy ^(12), (19), (20), (21)^. Based on findings after the Chernobyl accident, one study has suggested that polydactyly may be associated with radiation ^(22)^. However, the study after the Chernobyl accident did not consider any confounding factors ^(22)^. Yasuda et al. reported that no polydactyly/syndactyly infants were born to pregnant women in Fukushima Prefecture after the Fukushima Daiichi Nuclear Power Plant accident, who were exposed to external radiation doses of 2 mSv or higher ^(9)^. Rather, most polydactyly/syndactyly infants were born to pregnant women exposed to low doses of external radiation <1 mSV in Fukushima Prefecture ^(9)^. This indicates that it is extremely unlikely that radiation exposure is the cause of polydactyly in infants in the Fukushima Prefecture.

While the results need to be carefully interpreted because we were unable to perform a multivariate logistic regression analysis, as a complete separation of data points, amniotic cord syndrome was found to be more frequent in infants in the Fukushima RC than in the other 14 RCs. Unfortunately, the cause of amniotic cord syndrome is not known. Many cases seem to happen for no apparent reason ^(12), (23)^.

Therefore, the next research task would be to investigate each case in detail, including the possibility of familial inheritance, and also to clarify the reasons for the relatively high frequency of congenital anomalies in infants in Fukushima.

### Strengths

The Japanese congenital anomaly data published in the International Clearinghouse for Birth Defects Surveillance and Research (ICBDSR) did not include spontaneous abortion and induced abortion. Contrarily, the JECS data did include spontaneous abortion and induced abortion. ^(11), (24)^. Furthermore, whereas the JECS collects data from pregnant women who were managed in general hospitals, clinics, and maternity homes ^(11)^, the research facilities of the Japanese data of ICBDSR were mainly based on hospitals that provide care for women with high-risk pregnancies ^(11), (24)^. For these reasons, the JECS data are presumed to be closer to the actual frequency of congenital anomalies among the general population in Japan. In addition, the JECS is a prospective birth cohort study, and also has been conducted nationwide with the same protocol, further, collecting information on the basic attributes of the mothers of the infants ^(1), (2), (11)^. Therefore, we can say with certainty that there is no more suitable data beyond JECS to compare the frequency and risk for congenital anomalies in infants of Fukushima and other regions in Japan at this time.

### Limitations

First, JECS is a survey based on voluntary consent to participate. Not all pregnant women and their infants in Fukushima Prefecture and other regions participated in this survey ^(1), (2), (10)^. Therefore, there is a possibility of sample bias. Second, congenital anomalies were diagnosed up to one month of age. Third, detailed classification of congenital heart disease was not available. Fourth, we did not examine the data by region or year in Fukushima Prefecture. Finally, the JECS was designed primarily for examination of the relationship between environmental chemicals and children’s health. The analysis of the effects of radiation on pregnancy outcomes should be possible after the development of exposure-dose assessment and the consideration of statistical power to test the hypothesis, which will be taken into account in future research. Therefore, the association between radiation dose and pregnancy outcomes was not investigated in the present study.

### Conclusion

Overall, Fukushima Prefecture was found not to be an area at high risk for the occurrence of congenital anomalies in infants compared to other regions in Japan from 2011 to 2014.

## Article Information

### Conflicts of Interest

None

### Sources of Funding

This study was funded by the Ministry of the Environment, Japan. The findings and conclusions of this article are solely the responsibility of the authors and do not represent the official views of the above government.

### Acknowledgement

The authors are grateful to all the participants of the study. Members of the JECS group as of 2021 are as follows: Michihiro Kamijima (principal investigator, Nagoya City University, Nagoya, Japan); Shin Yamazaki (National Institute for Environmental Studies, Tsukuba, Japan); Yukihiro Ohya (National Center for Child Health and Development, Tokyo, Japan); Reiko Kishi (Hokkaido University, Sapporo, Japan); Nobuo Yaegashi (Tohoku University, Sendai, Japan); Koichi Hashimoto (Fukushima Medical University, Fukushima, Japan); Chisato Mori (Chiba University, Chiba, Japan); Shuichi Ito (Yokohama City University, Yokohama, Japan); Zentaro Yamagata (University of Yamanashi, Chuo, Japan); Hidekuni Inadera (University of Toyama, Toyama, Japan); Takeo Nakayama (Kyoto University, Kyoto, Japan); Hiroyasu Iso (Osaka University, Suita, Japan); Masayuki Shima (Hyogo Medical University, Nishinomiya, Japan); Hiroshige Nakamura (Tottori University, Yonago, Japan); Narufumi Suganuma (Kochi University, Nankoku, Japan); Koichi Kusuhara (University of Occupational and Environmental Health, Kitakyushu, Japan); and Takahiko Katoh (Kumamoto University, Kumamoto, Japan).

### Author Contributions

All authors confirm that they had full access to all the data in the study and they accept responsibility to submit it for publication. The authors’ contributions are as follows: H.N., K.F., M.H., T.N., K.S., S.Y., and K.H. designed the study. H.N., K.F., M.H., A.S., S.Y., and K.H. performed the study. H.N., and T.N. analysed the data. H.N., K.F., M.H., T.N., K.S., S.Y., and K.H. verified the underlying data. H.N., K.F., M.H., T.N., T.M., H.K., A.S., K.S., S.Y., and K.H. interpreted the findings. H.N., K.F., M.H., T.N., K.S., S.Y., and K.H. wrote the manuscript.

### Approval by Institutional Review Board (IRB)

The Ministry of the Environment’s Institutional Review Board on Epidemiological Studies (Reference no. 100910001).

### Informed Consent

Written informed consent was obtained from all participants.
